# Economic burden of resected squamous cell carcinoma of the head and neck in an incident cohort of patients in the UK

**DOI:** 10.1186/1758-3284-3-47

**Published:** 2011-10-28

**Authors:** Kun Kim, Mayur M Amonkar, Daniel Högberg, Frida Kasteng

**Affiliations:** 1 OptumInsight, Stockholm, Sweden; 2 GlaxoSmithKline, Collegeville, PA, USA; 3 i3 Innovus (currently OptumInsight), Stockholm, Sweden

**Keywords:** Squamous cell carcinoma of head and neck, Head and neck cancer, Oral cancer, Resection, Surgery, Cost of illness, Burden of illness

## Abstract

**Background:**

SCCHN is the sixth most common cancer worldwide. Locally advanced SCCHN continues to be a therapeutic challenge with high rates of morbidity and mortality and a low cure rate. Despite the apparent impact of SCCHN on patients and presumably society, the economic burden of the treatment of resected SCCHN patients in the UK has not been investigated.

**Methods:**

This retrospective data analysis was based on in- and outpatient care records extracted from Hospital Episode Statistic database and linked to mortality data in the UK. SCCHN patients with resection of lip, tongue, oral cavity, pharynx or larynx were followed for at least one year (max. of 5 years) from the date of first resection.

**Results:**

A total of 11,403 patients (mean age 63.2 years, 69.8% males) who met study criteria were followed for an average of 31 months. 32.3% of patients died in the follow-up period and the mean time to death was 16.9 months. In the first year, mean number of days of hospitalization and number of outpatient visits was 21.6 and 4.2, respectively; mean number of reconstructive and secondary surgeries was 0.32 and 0.14 per patient, respectively; 4.7% of the patients received radiotherapy and 12.2% received chemotherapy. From the second to fifth year healthcare utilizations rates were lower. Mean cost of post-operative healthcare utilization was £23,212 over 5 years (£19,778 for the first year and £1477, £847, £653 and £455 for years 2-5). Total cost of post-operative healthcare utilisation was estimated to be £255.5 million over the 5-year follow-up.

**Conclusions:**

In the UK, SCCHN patients after surgical resection needed considerable healthcare resources and incurred substantial costs. Study findings might provide a useful source for clinicians and decision makers in understanding the economic burden of managing SCCHN in the UK and also suggests a need for new therapies that could improve outcomes and reduce the disease burden.

## Background

Head and neck cancer (HNC) is a group of biologically similar cancers originating from the upper aerodigestive tract at various sites including the lips, oral cavity, nasal cavity, salivary glands, paranasal sinuses, thyroid, pharynx, and larynx. In the last 10 years, HNC has been one of the 10 most frequently diagnosed malignancies worldwide, with more than 640,000 people diagnosed and causing 7,500 deaths yearly [1]. In the UK, 7,538 people were newly diagnosed with HNC in 2006 and 2,594 people died from the disease in 2007 [2]. Recent trend studies reported that the incidence of HNC has risen over the last 20 years in the UK [3-7]. One of the findings showed that the incidence rate of oral and oropharyngeal cancer rose from 6.5 to 8.3 per 100,000 men and from 2.6 to 3.6 per 100,000 women between 1990--1999 [3]. According to a study of mortality in oral cancer in Europe, the age adjusted mortality rate in England and Wales was 2.7 and 1.05 per 100,000 inhabitants in men and women respectively and in Scotland the age adjusted mortality rate was 4.6 and 1.6 per 100,000 inhabitants in men and women, respectively between 1995--1999 [8]. In a projection of major cancers in the UK during 2006-2025, the mortality rate for oral cancer was estimated to still be growing in men and to be nearly constant in women [9].

The treatment of squamous cell carcinoma of the head and neck (SCCHN) is complex, partly because of the variety of tumor subsites and also because of the anatomic constraints of the head and neck region, together with the importance of maintaining organ function. Approximately 30--40% of patients with SCCHN present an early stage disease which is commonly managed by surgery (referred to as resection) or adjuvant radiotherapy with curative intent. For patients with advanced locoregionally disease without distant metastases, combinations of surgery, radiotherapy, and chemotherapy are used with the objective to maximize cure and maintain functional status through organ preservation. For patients with unresectable SCCHN, concurrent chemoradiotherapy is often chosen as a palliative treatment and may result in improved survival.

Although therapeutic advances have expanded treatment options, surgical resection is still considered an important treatment modality for SCCHN. The traditional treatment modality with surgical resection has a poor prognosis, 10--15% risk for local disease recurrence and 20--30% risk for disease progression within one year of treatment. According to a population-based study in Denmark, 1-year relative survival of patients with oral and pharynx cancer was 70% and 73% and the 5-year relative survival was 33% and 42% in men and women respectively [10]. Besides, surgery for oral cavity and oropharynx cancer was found to immediately cause impairment in physical, emotional and social functions and severely compromise the patient's quality of life (QoL) [11]. Overall QoL scores of patients with SCCHN were significantly worse at 3 and 6 months and returned to around preoperative scores at 12 months after treatment [12,13]. However, the patients with oral cancer, who were treated with surgery, did not restore preoperational level of function in appearance, swallowing, recreation, and chewing [14-16] and no significant overall improvement was found at 12 months among patients who underwent free-flap surgery or total laryngectomy [17-22]. Finally, the treatment is burdensome on those who survive. According to findings from registry data among SCCHN patients after treatment, 55% patients had a feeding tube placed, 13% needed a tracheotomy, 79% required opioid analgesics, and 78% had anti-emetics prescribed, and 34% reported grade 3--4 mucositis/stomatitis [23].

Despite the apparent impact of the disease on patients and presumably on society and health care, the economic burden of the treatment of resected SCCHN patients in the UK has not been investigated. Therefore, this study aimed at identifying the post-operative healthcare utilisation and its associated cost for an incident cohort of patients with SCCHN in the UK who underwent surgical resection.

## Methods

A retrospective analysis was performed by using Hospital Episode Statistic (HES) data, which covers all National Health Service (NHS) trusts and independent sectors in the UK. The scope of the data included secondary care facilities such as care trusts, mental health trust, NHS trust, ambulance trusts and emergency and urgent care but it did not include primary care facilities such as NHS direct/clinic centers, GP practices, dentists, opticians and pharmacists and also did not include accident and emergency care. The NHS data quality report concluded that HES data was a reliable source for activity-based analyses at national level in a provider's perspective based on high levels of completeness and validity for most fields related to patient identification in both inpatient and outpatient care [24]. However, the HES accident and emergency data still needs to be attested with reliability of the data [25] so it was not used in this analysis. The healthcare utilisation for patients in the HES data was identified with OPCS-4.5 codes which stores up individual procedures and interventions provided from inpatient and outpatient care. Mortality data between 2003 and 2009, provided by the Office for National Statistics (ONS), was used to identify deceased patients in the HES data. The requested data from HES and ONS were anonymized records, thus no patient's identity were disclosed for the purposes of this analysis.

**Figure 1:**
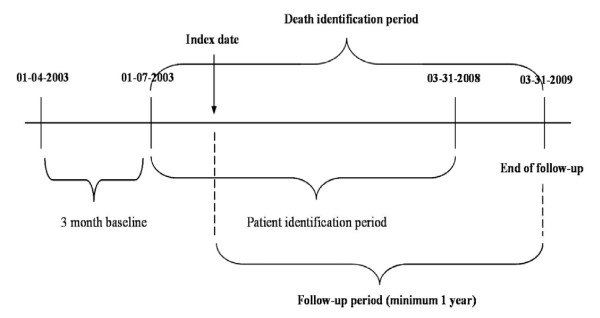
**Study observation period**.

The HES data was extracted from April 1, 2003 to March 31, 2009 and the data included patients who met the following inclusion criteria; evidence of cancer as primary diagnosis in lip, tongue, oral cavity, pharynx or larynx (ICD-10: C00--6, C09--10, C12--4, C32) and evidence of surgical resection from July 1, 2003 to March 31, 2008. The surgical resection was defined as including incision, excision, removal of foreign body, extirpation or -ectomy in respiratory tract, mouth, skin, soft tissue or bones and joints of skull of the patients. The selected patients were followed from the earliest date (index date) with the procedure code for primary surgical resection for at least 12 months or to the date of death or at the latest March 31, 2009. Patients who underwent resection between April 1, 2003 and June 30, 2003 were excluded to ensure that the patient would not be followed from a secondary resection. Patients under the age of 18 years were excluded. Figure 1 depicts the study observation period.

Post-operative healthcare utilization included the number of days of hospitalisation, the number of visits in outpatient care and treatments for chemotherapy-related adverse events. The chemotherapy-related treatments were identified by an episode of disease including anemia, neutropenia, thrombocytopenia, nausea, mucositis and pain rather than a specific treatment. The post-operative healthcare utilization also included the number of radiotherapy and chemotherapy sessions and secondary or reconstructive surgeries after resection. The secondary surgery was defined as resection following a primary resection. The reconstructive resection was defined as including plasty, plastic repair, reconstruction, and flap in respiratory tract, mouth, skin, soft tissue or bones and joints of skull of the patients.

The post-operative healthcare cost was measured by mapping the healthcare utilization to "the national schedule of reference costs 2008--09 for NHS Trusts" and "Unit costs of health & social care 2009" published by the Personal Social Services Research Unit (PSSRU). The unit costs for each procedure from inpatient and outpatient care are described in Additional file 1.

To account for the censored patients due to different lengths of follow-up in the data, the Kaplan-Meier sample-average (KMSA) method was employed. The KMSA is a technique to provide a consistent estimator of costs in the presence of censoring in the sample population[26]. The follow-up period was divided into each year and the mean cost of all uncensored patients during the each year was multiplied by the Kaplan-Meier estimate of the proportion alive at the beginning of the each year. Summing across these weighted costs for each interval resulted in the adjusted costs for censoring. The healthcare utilization and its costs were calculated in the first, second, third, fourth and fifth year from resection. The utilization and costs were to be interpreted as per year in the follow-up period for a group of the patients (incident cases) who were followed more than 1 year and less than 5 years.

## Results

**Figure 2:**
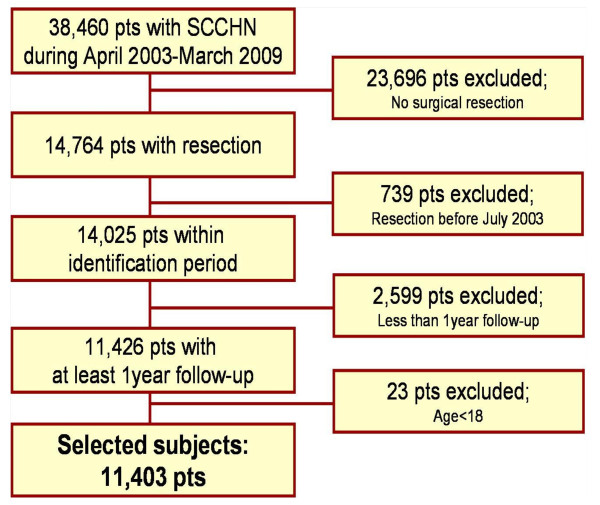
**Sample selection and attrition flow chart**.

A total of 38,460 patients who were diagnosed with SCCHN from April 1, 2003 to March 31, 2009 were extracted from the HES data. 14,764 patients (38.4%) underwent a surgical resection. Among the resected patients, we excluded 739 patients who underwent resection between April 1 and June 30, 2003. 2,599 patients who underwent resection after March 31, 2008 were also excluded as they had less than 12 months follow-up from the first resection. After excluding a further 23 patients, who were under the age of 18 years, 11,403 patients were finally included in the sample population. Figure 2 presents the flow chart for selection of the incident cohort of patients included in this study.

### Patient Cohort Characteristics

The mean age of the patient cohort for this study was 63.2 years (SD 12.6) and 69.8% of patients were men. The mean age, the ratio of men to women and the proportion of age groups in the population corresponded to UK incidence statistics for HNC reported by Cancer Research UK [27,28]. Lip cancer patients were the oldest (mean 69.6 years) compared to the other four cancer sites while the larynx cancer subgroup had the largest percentage of males (85%). The mean length of follow-up was 31.0 months (SD 18.4). The HES data were extracted mainly from healthcare facilities in England. A majority of the patients in the population came from England and there were a minority from Scotland, Wales and Northern Ireland. Larynx cancer patients accounted for 29% and pharynx cancer patients for 20% of the cohort. Oral cavity, tongue and lip cancers patients accounted for 21%, 19% and 11%, respectively (Table 1). A total of 3,686 deaths were identified during follow-up and the mean length from first resection to death was 16.9 months. 46.3% of the deaths occurred within the first year of follow up with the highest and lowest percentage of deaths occurring with the pharyngeal (47.9%) and lip cancer (31.5%) cohorts, respectively. The highest percentage of deaths occurred among larynx cancer patients (37.3%), followed by pharynx (35.4%), oral cavity (32.7%), tongue (30.3%) and lip cancer patients (16.2%) (Table 2).

**Table 1:** Patient cohort characteristics

Cancer site	Lip		Tongue		Oral cavity		Pharynx		Larynx		SCCHN	
	N	%	N	%	N	%	N	%	N	%	N	%
	1,250	11.0	2,167	19.0	2,372	20.8	2,304	20.2	3,310	29.0	11,403	100.0
Gender (Male)	726	58.1	1,267	58.5	1,436	60.5	1,717	74.5	2,813	85.0	7,959	69.8

Age group												
18-44	72	5.8	213	9.8	159	6.7	230	10.0	86	2.6	760	6.7
45-64	328	26.2	1,062	49.0	1,221	51.5	1,459	63.3	1,499	45.3	5,569	48.8
65+	849	67.9	892	41.2	991	41.8	615	26.7	1,725	52.1	5,072	44.5

Geographic location												
North East	96	7.7	109	5.0	167	7.0	160	6.9	280	8.5	812	7.1
North West	214	17.1	332	15.3	447	18.8	399	17.3	548	16.6	1,940	17.0
Merseyside	0	0.0	0	0.0	0	0.0	0	0.0	0	0.0	0	0.0
Yorkshire and Humber	121	9.7	225	10.4	276	11.6	256	11.1	375	11.3	1,253	11.0
East Midlands	112	9.0	212	9.8	245	10.3	244	10.6	269	8.1	1,082	9.5
West Midlands	92	7.4	206	9.5	228	9.6	170	7.4	300	9.1	996	8.7
East of England	147	11.8	204	9.4	185	7.8	207	9.0	304	9.2	1,047	9.2
London	134	10.7	280	8.3	291	12.3	269	11.7	431	13.0	1,305	11.4
South East	169	13.5	338	15.6	279	11.8	289	12.5	437	13.2	1,512	13.3
South West	156	12.5	225	10.4	209	8.8	272	11.8	302	9.1	1,164	10.2
Scotland	1	0.1	4	0.2	4	0.2	2	0.1	0	0.0	11	0.1
No fixed above	0	0.0	2	0.1	0	0.0	3	0.1	2	0.1	7	0.1
Wales	6	0.5	9	0.4	12	0.5	16	0.7	13	0.4	56	0.5
Foreign (including Isle of Man and Channel Islands)	1	0.0	18	0.8	21	0.9	14	0.6	40	1.2	93	0.8
Unknown	1	0.0	3	0.1	8	0.3	3	0.1	8	0.2	22	0.2
Northern Ireland	0	0.0	0	0.0	0	0.0	0	0.0	1	0.0	1	0.0

	Mean	SD	Mean	SD	Mean	SD	Mean	SD	Mean	SD	Mean	SD

Age	69.6	13.9	61.8	13.7	62.8	12.5	58.3	11.1	65.4	10.7	63.2	12.6

Length of follow-up (months)	34.9	16.9	31.4	18.3	30.8	18.5	30.1	18.3	30	18.7	31	18.4

^1 ^months

**Table 2:** Number of deaths identified by year and cancer type

Cancer site	Lip		Tongue		Oral cavity		Pharynx		Larynx		SCCHN	
	(N = 1,250)		(N = 2,167)		(N = 2,372)		(N = 2,304)		(N = 3,310)		(N = 11,403)	
Length of time to death^1,2^	Mean	SD	Mean	SD	Mean	SD	Mean	SD	Mean	SD	Mean	SD
	22.4	15.3	16.4	13.3	17.4	14.1	16.4	13.4	16.4	13.6	16.9	13.8

Number of deaths^3^	N	%	N	%	N	%	N	%	N	%	N	%
	203	16.2	656	30.3	776	32.7	815	35.4	1,236	37.3	3,686	32.3
1 year	64	31.5	310	47.3	360	46.4	390	47.9	582	47.1	1,706	46.3
2 year	60	29.6	197	30.0	213	27.4	230	28.2	344	27.8	1,044	28.3
3 year	36	17.7	82	12.5	105	13.5	117	14.4	180	14.6	520	14.1
4 year	27	13.3	47	7.2	65	8.4	51	6.3	84	6.8	274	7.4
5 year	16	7.9	20	3.0	33	4.3	27	3.3	46	3.7	142	3.9

^1 ^months; ^2 ^among the deceased patients; ^3 ^for year wise-breakdown %s are based on number of deaths within a category

### Estimation of survival over 5-year follow-up

To calculate the total cost of the post-operative healthcare utilisation over the 5-year follow-up, the number of surviving patients during the 5-year follow-up was estimated, based on the number of followed patients and the number of deaths in the population. The number of surviving patients was assumed to be equal to the number of patients surviving the previous year deducted by the number of deaths among both the followed patients and the censored patients at the previous year. The number of deaths among the censored patients was estimated by the proportion of deaths in the followed patients multiplied with the number of the censored patients. At the beginning of the follow-up, there were 11,403 patients in the cohort. Since all subjects were followed at least 1 year, there was no censored patient and there were 9,697 surviving patients at the beginning of the second year in the cohort. The number of surviving patient per the rest of year was estimated as described (Table 3). The total cost of the post-operative healthcare utilisation was calculated by multiplying the mean costs in each year with the number of surviving patients in each year.

**Table 3:** Total number of deaths and the estimation of survived patients by year

Year	No. of patient followed	No. of deaths identified	No. of patient censored per year^1^	Mortality rate per year^2^	No. of death among censored patients^3^	No. of survived patient estimated by KMSA^4 ^	Cumulative survival, all-causes (%)
1 year	11,403	1,706	0	15.0%	0	11,403	-
2 year	9,697	1,044	2,046	10.8%	220	9,697	85.0%
3 year	6,607	520	1,767	7.9%	139	8,433	74.0%
4 year	4,320	274	1,592	6.3%	101	7,774	68.2%
5 year	2,454	-	-	-	-	7,399	64.9%

^1^= No. of followed patient - No. of followed patient at next year - No. of death

^2 ^= No. of death/No. of followed patient

^3 ^= No. of censored patient * mortality rate per year

^4 ^= No. of patient at previous year - No. of death at previous year - No. of death among censored patient at previous year

### Post-operative healthcare utilisation

**Figure 3:**
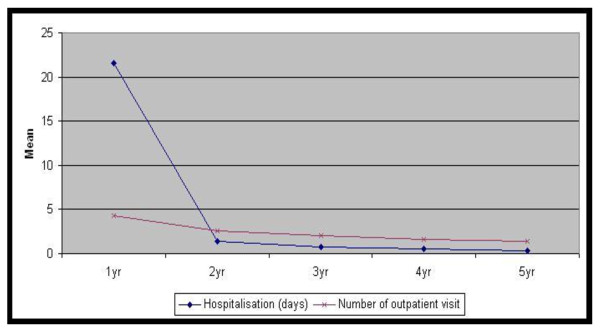
**Mean days of hospitalisation and mean number of outpatient visit after surgery over 5 years**. Year 1 yr 2 yr 3 yr 4 yr 5 yr. Hospitalisation (days) 21.60 1.38 0.70 0.50 0.30. Number of outpatient visit 4.25 2.54 1.99 1.66 1.43.

**Figure 4:**
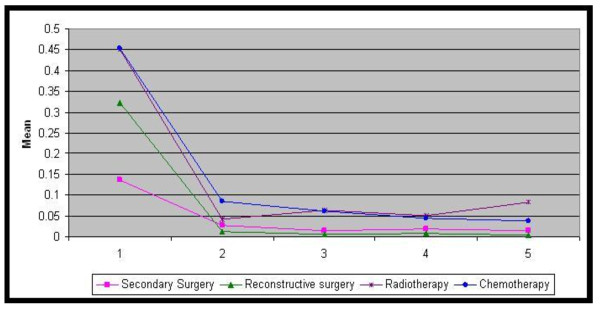
**Mean number of healthcare utilisation procedures after surgery over 5 years**. Year 1 yr 2 yr 3 yr 4 yr 5 yr. Secondary Surgery 0.137 0.028 0.016 0.020 0.015. Reconstructive surgery 0.322 0.012 0.007 0.008 0.005. Radiotherapy 0.451 0.043 0.065 0.052 0.084. Chemotherapy 0.452 0.085 0.062 0.045 0.038.

In the first year, the mean number of days of hospitalisation was 21.6 and the mean number of outpatient visits was 4.2 in the population. The number of reconstructive surgeries was 0.32 per patient and the number of secondary surgeries was 0.14 per patient. 4.7% of the patients received radiotherapy and 12.2% of the patients received chemotherapy in either an inpatient or outpatient setting. There were a total of 5,152 radiotherapy sessions and 5,153 chemotherapy sessions in inpatient and outpatient care. Half of the patients treated with chemotherapy were admitted due to chemotherapy-related conditions (Figure 3, 4).

From the second year, healthcare utilisation was rapidly reduced and the difference in healthcare utilisation from the second year to fifth year was small. From the second to fifth year, the mean number of days of hospitalisation was 0.3--1.4 and the mean number of outpatient visit was 1.4--2.5. The number of reconstructive surgery was 0.005--0.012 per patient and the number of secondary surgeries was 0.015--0.028 per patient. The mean number of radiotherapy sessions was 0.04--0.08 per patient and the number of chemotherapy sessions was 0.04--0.09 per patient (Figure 3, 4).

### Post-operative healthcare utilisation cost

Most of the costs were related to inpatient care and inpatient stay was the main cost driver over the 5 year follow-up. For the five years, cost of inpatient care accounted for 94.7% of the total cost. The inpatient care cost constituted of hospitalization cost (84.8%) and reconstructive surgery (11.4%), followed by secondary surgery (1.5%), radiotherapy (0.9%) and chemotherapy (0.5%). Outpatient visits accounted for 94.6% of outpatient care cost and cost for radiotherapy and chemotherapy accounted for 4.7% and 0.7% of outpatient care cost, respectively. The mean cost of post-operative healthcare utilisation for the resected patients with SCCHN was £23,212 over 5 years. The mean cost per year was £19,778 for the first year, £1,477 for second year, £847 for third year, £653 for fourth year and £455 for the fifth year (Table 4). The post-operative treatment for larynx cancer was most costly, £28,981 over 5 years, followed by pharynx cancer (£25,827), oral cavity cancer (£25,311), tongue cancer (£19,493) and lip cancer (£5,790) (Table 5). The total cost of the post-operative healthcare utilisation for the cohort of resected SCCHN was estimated to be £255.5 million over the 5-year follow-up.

**Table 4:** Mean cost of post-operative treatment for resected SCCHN patient per year

Followed year after resection	1 yr	2 yr	3 yr	4 yr	5 yr	Grand total
Number of patients	11,403	9,697	8,433	7,774	7,399	44,706
Secondary Surgery	208	45	27	29	19	328
Reconstructive surgery	2,275	85	49	55	38	2,502
Radiotherapy	187	4	6	1	2	201
Chemotherapy	67	17	10	11	8	113
Chemo related condition						
Anemia	33	6	3	2	1	45
Neutropenia	36	6	3	2	3	49
Thrombocytopenia	37	6	3	2	0	48
Nausea/Emesis	14	1	1	1	1	18
Mucositis	5	0	0	0	1	6
Pain	19	3	3	0	0	26
	
	144	23	13	7	5	192
Inpatient stay	16,448	1,050	536	378	230	18,642
**Inpatient care cost (£)**	**19,330**	**1,224**	**641**	**482**	**302**	**21,978**

Outpatient visit						
General surgery	16	15	16	13	13	73
Oral surgery	244	128	94	75	63	603
Restorative dentistry	24	25	17	16	13	95
Oral and maxillo facial surgery	27	20	16	16	15	94
Plastic surgery	11	6	5	5	4	31
Pain management	0	0	0	0	0	0
General medicine	27	23	23	20	16	109
Palliative medicine	14	12	9	5	8	48
Medical oncology	50	20	17	13	10	111
Radiology	1	0	0	0	0	2
	
	414	249	198	163	143	1,167
Radiotherapy	34	4	7	5	8	57
Chemotherapy	1	0	1	3	3	9
**Outpatient care cost (£)**	**448**	**254**	**206**	**172**	**153**	**1,233**

**Total cost (£)**	**19,778**	**1,477**	**847**	**653**	**455**	**23,212**

**Table 5:** Mean cost of post-operative treatment for resected SCCHN patient by cancer site

Followed year after resection	Lip	Tongue	Oral cavity	Pharynx	Larynx	SCCHN
Number of patients	1,250	2,167	2,372	2,304	3,310	11,403
Secondary Surgery	176	328	318	185	489	328
Reconstructive surgery	2,368	2,152	5,411	2,283	843	2,502
Radiotherapy	38	203	183	325	189	201
Chemotherapy	13	110	82	217	92	113
Chemo related condition						
Anemia	4	45	53	71	37	45
Neutropenia	6	78	32	101	23	49
Thrombocytopenia	5	56	48	82	37	48
Nausea/Emesis	3	16	15	43	10	18
Mucositis	3	6	14	9	1	6
Pain	2	29	26	28	32	26
	
	23	230	188	333	139	192
Inpatient stay	2,180	14,888	16,961	21,424	26,691	18,642
**Inpatient care cost (£)**	**4,798**	**17,910**	**23,143**	**24,766**	**28,443**	**21,978**

Outpatient visit						
General surgery	87	66	78	61	77	73
Oral surgery	508	959	1,337	337	55	603
Restorative dentistry	38	88	206	116	29	95
Oral and maxillo facial surgery	68	167	200	52	9	94
Plastic surgery	124	22	21	22	14	31
Pain management	0	0	0	0	0	0
General medicine	107	98	101	99	130	109
Palliative medicine	9	43	39	91	45	48
Medical oncology	38	90	94	164	129	111
Radiology	1	2	2	3	1	2
	
	980	1,535	2,078	943	490	1,167
Radiotherapy	11	46	80	107	34	57
Chemotherapy	1	2	9	11	14	9
**Outpatient care cost (£)**	**992**	**1,583**	**2,168**	**1,061**	**538**	**1,233**

**Total cost (£)**	**5,790**	**19,493**	**25,311**	**25,827**	**28,981**	**23,212**

## Discussion

The findings in the analysis were based on a large cohort of patients who were resected for SCCHN between 2003 and 2009. The patient group seemed representative of the resected SCCHN population in the UK. Rogers et al., estimated a 5 year survival rate by the Kaplan-Meier survival analyses among patients who underwent primary surgery for oral cancer during 1992--2002 in the UK [29]. Among the patients during 2000--2 in the study, overall survival rates at 2 year and 5 year were 74% and 64%, respectively. In the study cohort, overall survival rates at 2 year and 5 year were 74.0% and 64.9%, respectively, and among the resected oral cancer patients (tongue and oral cavity cancer only), overall survival rates at 2 year and 5 year were 74.2% and 65.7%, respectively. According to a comparative report of Scottish and English cohorts, a treatment modality involving surgery for HNC treatment accounted for 51.3% and 51.6% in both cohorts [30]. Among the 38,460 patients with SCCHN extracted from HES data, 14,764 patients (38.4%) of them were identified to undergo resection. The difference might be caused by miscellaneous surgeries such as biopsy that was not regarded as surgical resection in the sample selection.

In 2001, van Agthoven reported that the average cost per patient was €21,858 for HNC and €27,629 for the recurrent disease for two years in the Netherlands[31]. Based on 36% of the relapse rate from a prospective cohort study of patients with SCCHN [32], the weighted average cost would be €23,936 and it could be converted to £23,066 in 2009 (1 Euro = 0.89 GBP) [33]. It was comparable to sum of mean cost per first and second year in the results which was £21,255. Another study reported that the cost of a major head and neck surgical case was £32,000 in the UK in 2007, including inpatient cost, resection/reconstructive surgery and also nursing cost, intensive therapy unit, rehabilitation and support service cost which were not included in this analysis [34].

One of the methodological difficulties of using the HES data was that the outpatient care records had limited levels of completeness in procedural and diagnostic recording. Healthcare providers in the UK are required to submit their patient's records to NHS on a regular basis. Mandatory fields in the data submission such as patient identification details and specialty of outpatient care typically achieved a high level of completeness but optional fields still needed to be improved [24]. According to the HES outpatient data quality report [35], 97% of primary diagnosis were recorded as unknown and 93% of the main procedure/intervention was recorded as "not known" from the outpatient record in 2006--07. Thus, outpatient visit for radiotherapy and chemotherapy might be underestimated where "not known" was recorded for radiotherapy and chemotherapy-related procedures. On the other hand, the mean number of outpatient visit per year seemed to be consistent with other studies. According to a recent study of the trend of follow-up for patients with HNC in the UK, the number of follow-up appointments for patients were reported to once every 3--6 months in the third year and once every 6--12 months in the fourth and fifth year [36] and the findings were similar with the frequency of the outpatient visit identified in the sample population.

## Conclusion

This retrospective data analysis was performed and based on a large number of empirical patient records from in- and outpatient care, and the study population seemed to be representative for resected SCCHN patients in the UK. Almost 38% of SCCHN patients underwent surgical resection and the resected patients had a high mortality rate. The patients with SCCHN needed considerable healthcare resources after surgical resection, associated with substantial costs. Over the 5-year follow-up period, 85% of costs occurred in the first year following the first surgical resection. Since outpatient-based radiotherapy and chemotherapy utilisation seemed to be underestimated, the outpatient cost of post-operative healthcare utilisation was likely to be even higher. Nevertheless, the average total cost of post-operative healthcare utilisation appeared to be comparable to previous studies.

These findings might provide a useful source for clinicians and decision makers in understanding the economic burden of managing SCCHN in the UK and also suggests a need for new therapies that could improve outcomes and reduce the disease burden.

## List of abbreviations

HNC: Head and Neck Cancer; HES: Hospital Episode Statistic; KMSA: the Kaplan-Meier Sample-Average; NHS: National Health Service; ONS: the Office for National Statistics; PSSRU: the Personal Social Services Research Unit; QoL: Quality of Life; SCCHN: Squamous Cell Carcinoma of the Head and Neck.

## Competing interests

This analysis was financially supported by GlaxoSmithKline. Mayur Amonkar is an employee of GlaxoSmithKline.

## Authors' contributions

KK has made contributions to the study design, analysis and interpretation of data and drafting and revising the manuscript. MMA has been in involved in conception and design of the study. DH participated in drafting and revising the manuscript. FK has contributed to conception and design of the study. All authors have read and approved the final manuscript.

## Supplementary Material

Additional file 1 **Unit costs for procedures from inpatient and outpatient care**. The unit costs for the post-operative healthcare cost were based on "the national schedule of reference costs 2008--09 for NHS Trusts" and "Unit costs of health & social care 2009 " published by PSSRU. Click here for file
