# Supplementary material for: Economic burden of resected squamous cell carcinoma of the head and neck in an incident cohort of patients in the UK
Source: Head Neck Oncol. 2011 Oct 28;3:47. doi: 10.1186/1758-3284-3-47 (PMC3219567; doi:10.1186/1758-3284-3-47)
Supplement: Additional file 1 — Unit costs for procedures from inpatient and outpatient care. The unit costs for the post-operative healthcare cost were based on "the national schedule of reference costs 2008--09 for NHS Trusts" and "Unit costs of health & social care 2009 " published by PSSRU. [file 1758-3284-3-47-S1.DOC]

| **Description** | **Unit Cost** | |
| --- | --- | --- |
| *Inpatient stay (per day)* |  |  |
| Other Neoplasms with length of stay 1 day or more with/without CC^1^ | £761 | - |
| *Secondary surgery* |  |  |
| Minor Mouth or Throat Procedures 19 years and over with/without CC^1,2^ | £570 | - |
| Intermediate Mouth or Throat Procedures 19 years and over with/without Major/Intermediate CC^1,2^ | £859 | - |
| Complex Major Mouth or Throat Procedures with/without Major/Intermediate CC^1,2^ | £2,933 | - |
| Minor Maxillo-facial Procedures with/without CC^1,2^ | £1,092 | - |
| Intermediate Maxillo-facial Procedures 19 years and over with/without CC^1,2^ | £1,533 | - |
| Major Maxillo-facial Procedures 19 years and over^1,2^ | £2,220 | - |
| Complex Major Maxillo-facial Procedures^1,2^ | £2,225 | - |
| Minor Head, Neck and Ear Disorders 19 years and over with/without CC^1,2^ | £404 | - |
| *Reconstructive* |  |  |
| Reconstruction Procedures Category 6 with/without CC^1,2^ | £11,367 | - |
| Reconstruction Procedures Category 4^1,2^ | £9,518 | - |
| Reconstruction Procedures Category 2^1,2^ | £7,647 | - |
| Reconstruction Procedures Category 1 19 years and over^1,2^ | £4,562 | - |
| *Chemotherapy related conditions* |  |  |
| Anemia^1,2^ | £973 | - |
| Neutropenia^1,2^ | £3,270 | - |
| Thrombocytopenia^1,2^ | £1,252 | - |
| Nausea/Emesis^1,2^ | £629 | - |
| Mucositis^1,2^ | £741 | - |
| Pain^1,2^ | £2,127 | - |
| Radiotherapy | Inpatient | outpatient |
| Deliver a fraction of Total Body Irradiation^3,4^ | £918 | £597 |
| Prepare for Total Body Irradiation^3,4^ | £1,841 | £2,433 |
| Prepare for Intracavitary Radiotherapy^3,4^ | £735 | £397 |
| Prepare for Interstitial Radiotherapy^3,4^ | £932 | £1,322 |
| Other Radiotherapy Planning^3,4^ | £1,055 | £271 |
| Deliver a fraction of treatment on a superficial or orthovoltage machine^3,4^ | £568 | £82 |
| Deliver a fraction of Intracavitary Radiotherapy with/without General  Anaesthetic^3,4^ | £1,879 | £448 |
| Deliver a fraction of Interstitial Radiotherapy^3,4^ | £1,894 | £1,580 |
| Other Radiotherapy Treatment^3,4^ | £2,170 | £134 |
| *Chemotherapy* |  |  |
| Procure Chemotherapy drugs for regimens in Band 1^5,6^ | £231 | £198 |
| Procure Chemotherapy drugs for regimens in Band 2^5,6^ | £386 | £303 |
| Procure Chemotherapy drugs for regimens in Band 3^5,6^ | £493 | £612 |
| Procure Chemotherapy drugs for regimens in Band 4^5,6^ | £612 | £625 |
| Procure Chemotherapy drugs for regimens in Band 5^5,6^ | £812 | £806 |
| Procure Chemotherapy drugs for regimens in Band 6^5,6^ | £1,004 | £928 |
| Procure Chemotherapy drugs for regimens in Band 7^5,6^ | £1,122 | £911 |
| Procure Chemotherapy drugs for regimens in Band 8^5,6^ | £1,065 | £1,627 |
| Procure Chemotherapy drugs for regimens in Band 9^5,6^ | £1,256 | £2,030 |
| Procure Chemotherapy drugs for regimens in Band 10^5,6^ | £1,773 | £2,280 |
| Deliver exclusively Oral Chemotherapy^5,6^ | £158 | £209 |
| Deliver simple Parenteral Chemotherapy at first attendance^5,6^ | £180 | £272 |
| Deliver more complex Parenteral Chemotherapy at first attendance^5,6^ | £213 | £447 |
| Deliver complex Chemotherapy, including prolonged infusional treatment  at first attendance^5,6^ | £259 | £335 |
| Deliver subsequent elements of a Chemotherapy cycle^5,6^ | £251 | £227 |
| Deliver chemotherapy for regimens not on the national list^5,6^ | £258 | £96 |
| Intravenous induction of labour^7^ | £11 | £11 |
| Intravenous chemotherapy^7^ | £11 | £11 |
| Intravenous immunotherapy^7^ | £11 | £11 |
| Intravenous injection of non radioactive diagnostic substance^7^ | £11 | £11 |
| Intravenous injection of antimicrobial therapy^7^ | £11 | £11 |
| Other specified other intravenous injection^7^ | £11 | £11 |
| Unspecified other intravenous injection^7^ | £11 | £11 |
| Outpatient visit | First appointment | Subsequent appointment |
| General surgery^8,9^ | £145 | £97 |
| Oral surgery^8,9^ | £123 | £86 |
| Restorative dentistry^8,9^ | £100 | £87 |
| Oral and maxillo facial surgery^8,9^ | £145 | £97 |
| Plastic surgery^8,9^ | £112 | £77 |
| Pain management^8,9^ | £163 | £103 |
| General medicine^8,9^ | £190 | £130 |
| Palliative medicine^8,9^ | £422 | £307 |
| Medical oncology^8,9^ | £179 | £121 |
| Radiology (radiographer)^10^ | £43 | £43 |

^1^National Schedule of Reference Costs Year: '2008—09' - NHS Trusts Non-Elective Inpatient (Long Stay) HRG Data

^2^National Schedule of Reference Costs Year: '2008—09' - NHS Trusts Non-Elective Inpatient (Short Stay) HRG Data

^3^National Schedule of Reference Costs Year: '2008—09' - NHS Trusts Radiotherapy: Inpatients

^4^National Schedule of Reference Costs Year: '2008—09' - NHS Trusts Radiotherapy Planning: Outpatients

^5^National Schedule of Reference Costs Year: '2008—09' - NHS Trusts Chemotherapy Procurement: Inpatient

^6^National Schedule of Reference Costs Year: '2008—09' - NHS Trusts Chemotherapy Procurement: Outpatients

^7^Labour cost for nurse for 30min was used in PSSRU 2009 (Table 12.4 and 12.3)

^8^National Schedule of Reference Costs Year: '2008—09' - Consultant Led: First Attendance Non-Admitted Face to Face

^9^National Schedule of Reference Costs Year: '2008—09' - Consultant Led: Follow up Attendance Non-Admitted Face to Face

^10^Cost for radiographer per hour of client contact in PSSRU 2009 (Table 11.5)
